# The dynamics of experiencing Gestalt and Aha in cubist art: pupil responses and art evaluations show a complex interplay of task, stimuli content, and time course

**DOI:** 10.3389/fpsyg.2024.1192565

**Published:** 2024-03-13

**Authors:** Blanca T. M. Spee, Jozsef Arato, Jan Mikuni, Ulrich S. Tran, Matthew Pelowski, Helmut Leder

**Affiliations:** ^1^Vienna Cognitive Science Hub, University of Vienna, Vienna, Austria; ^2^Department of Cognition, Emotion, and Methods in Psychology, Faculty of Psychology, University of Vienna, Vienna, Austria; ^3^Department of Neurology, Center of Expertise for Parkinson & Movement Disorders, Radboud University Medical Center, Donders Institute for Brain, Cognition and Behavior, Nijmegen, Netherlands

**Keywords:** art research, pupillometry, Gestalt perception, Aha moment, empirical art and aesthetics

## Abstract

**Introduction:**

Gestalt perception refers to the cognitive ability to perceive various elements as a unified whole. In our study, we delve deeper into the phenomenon of Gestalt recognition in visual cubist art, a transformative process culminating in what is often described as an Aha moment. This Aha moment signifies a sudden understanding of what is seen, merging seemingly disparate elements into a coherent meaningful picture. The onset of this Aha moment can vary, either appearing almost instantaneously, which is in line with theories of hedonic fluency, or manifesting after a period of time, supporting the concept of delayed but more in-depth meaningful insight.

**Methods:**

We employed pupillometry to measure cognitive and affective shifts during art interaction, analyzing both maximum pupil dilation and average dilation across the trial. The study consisted of two parts: in the first, 84 participants identified faces in cubist paintings under various conditions, with Aha moments and pupil dilation measured. In part 2, the same 84 participants assessed the artworks through ratings in a no-task free-viewing condition.

**Results:**

Results of part 1 indicate a distinctive pattern of pupil dilation, with maximum dilation occurring at both trial onset and end. Longer response times were observed for high-fluent, face-present stimuli, aligning with a delayed but accurate Aha-moment through recognition. Additionally, the time of maximum pupil dilation, rather than average dilation, exhibited significant associations, being later for high-fluent, face-present stimuli and correct detections. In part 2, average, not the time of maximum pupil dilation emerged as the significant factor. Face-stimuli and highly accessible art evoked stronger dilations, also reflecting high clearness and negative valence ratings.

**Discussion:**

The study underscores a complex relationship between the timing of recognition and the Aha moment, suggesting nuanced differences in emotional and cognitive responses during art viewing. Pupil dilation measures offer insight into these processes especially for moments of recognition, though their application in evaluating emotional responses through artwork ratings warrants further exploration.

## 1 Introduction

Gestalt perception, the ability to discern meaningful structures by organizing sensory data, is an essential facet of our interactions with the world (Wertheimer, 1923; Van de Cruys and Wagemans, 2011a; Wagemans et al., 2012). The concept is deeply entrenched in the field of visual art, particularly in non-representational art styles such as Cubism, where abstract, fragmented forms coalesce into a holistic image (Arnheim, 1954). This transformative moment is often related to the ‘Aha moment’ (Topolinski and Reber, 2010; Muth and Carbon, 2013).

Aha moments, also referred to as ‘epiphany’, are moments of sudden realization, insight, or comprehension. The term is often used in psychology and cognitive science to describe the instance at which an individual moves from not understanding or being unable to solve a problem to sudden comprehension (Muth and Carbon, 2013; Danek and Wiley, 2017). It can also refer to the moment of recognition when one sees the solution to a problem, the answer to a question, or the meaning behind a complex pattern. Aha moments are pivotal points in our perceptual experience of art. They can be seen as the junctures where the veil of abstraction and ambiguity is lifted, and the true essence—or the personal meaning—of an artwork is revealed.

In our study, we are particularly interested in the temporal aspect of these Aha moments—the ‘when’ of their occurrence—which is a yet underexplored facet in the research of art interaction. The unique qualities of the Aha moment, particularly its suddenness (Gick and Lockhart, 1995; Kounios and Beeman, 2014), and the ease or difficulty with which this moment of recognition arrives (Topolinski and Reber, 2010), form the crux of our research investigation. Specifically, we are interested in understanding the appearance of slow and fast processes of Gestalt recognition, corresponding with an early or delayed Aha moment, respectively. With these concepts in mind, our primary research question asks: “How do timing and success in Gestalt recognition, leading to Aha moments, depend on the accessibility of the stimulus, and how does this influence artwork evaluation?”

Previous art research posits that both quick and slow recognition processes exist. Quick Gestalt recognition is often associated with fluent processing, marked by immediate ease and appreciation of perceptual fluency (Reber et al., 2004; Belke et al., 2010a). This ‘hedonic fluency effect’ proposes that easily recognized images generate more positive emotions and are more liked. Conversely, slower recognition processes suggest that viewers derive reward from the effort invested in decoding abstract patterns in visual art, followed by a delayed ease, leading to profound insight and appreciation (Van de Cruys and Wagemans, 2011a,b; Wagemans et al., 2012; Wagemans, 2013). These research findings suggest that art perception, in terms of Gestalt recognition and Aha moments, is not a straightforward process but instead, a dynamic interplay between fast and slow processes, regulated by both top-down and bottom-up cognitive processes that are governed by the artwork’s accessibility and the viewer’s cognitive and emotional engagement (Leder et al., 2004; Leder and Nadal, 2014).

Our study seeks to examine this interplay by evaluating the impact of task specification (recognition task in a public or private condition versus free-viewing), stimulus content (faces versus landscapes), and ease of recognition (accessibility). In addition, we also implement and test an implicit physiological measure—pupillometry—to capture the moment and measuring, if behavioral and physiological responses coincide (Laeng et al., 2012; Mathôt, 2018).

Our experiment is divided into two parts. Part 1 involves eliciting Aha moments through Gestalt recognition of faces in cubist art, using varying accessibility (high = easy/fluent, medium, low = difficult/non-fluent) stimuli, and recording response times. In addition to stimulus content and accessibility, a key experimental manipulation is the introduction of a public versus private paradigm to amplify the effort motivation expended during the recognition task. In part 1, we examine the possible scenarios for response times, including a shorter response time indicative of perceptual fluency versus a longer response time suggestive of a slower mechanism involved in Gestalt recognition; for the latter, we argue that this process is top-down steered to ensure accuracy of Gestalt detection and Aha as a meaningful insight into Gestalt recognition. We hypothesize that a recognition-oriented task will necessitate more time and effort for face recognition, indicating slower recognition processes, which is enhanced in the public condition. In contrast, we expect faster response times for non-face stimuli like landscapes, suggestive of a superficial, non-accurate immediate response. We will further explore if the performance outcome itself, that is, if the person made a correct answer/hit or an error (false alarm or missed target), is associated with response times.

In line with these predictions and to underpin our findings, we will utilize pupillometry, a well-established method to study sudden shifts in cognitive and affective quality. We anticipate finding associations between the time of the maximum pupil dilation and our main predictors, stimulus content and accessibility, as previous studies have shown (Kuchinke et al., 2009; Elschner et al., 2018), serving as a physiological marker for gaining Aha through Gestalt recognition (Laeng et al., 2012; Mathôt, 2018). Hereby, we will also investigate the influence of our newly introduced effort motivation to gain a deeper understanding of pupillometric evidence supporting Aha detection in art research.

However, an issue with pupillometry is that it has been used for moments of shifts concerning recognition and detecting emotions. Hence, it is not yet clear if pupil dilations rather state recognizing patterns or are an emotional response. Therefore, in part 2 of our study, we will allow free viewing of the artworks without a specific task to investigate if pupil dilations respond differently, delivering a clearer interpretation of pupil dilations as a physiological marker. Here, we hope to observe a correlation between average pupil dilations and our main factors, which are stimulus content and accessibility, as a reflection of the emotional response toward the artworks. We hypothesize that compared to part 1 in part 2, the time of maximum pupil dilation might be less consistent or significant, that is, showing maximum dilations at different time points during the trial.

Building on prior work (Kuchinke et al., 2009; Elschner et al., 2018), we will consider a range of ratings for aesthetic judgments and attributes (arousal, clearness, liking, complexity, comprehension, and emotional valence) after image presentation in part 2. Despite a general analysis, where we anticipate positive associations across these attributes with face stimuli and varying levels of fluency, we will also investigate associations with pupil dilations. Here, we specifically expect positive associations with arousal, comprehension, clearness, liking, and positive valence. Furthermore, we will examine how performance in part 1 influences part 2’s subjective ratings, as cognitive processing theories suggest that the depth of our processing and comprehension can affect our appraisal of an artwork (Leder and Nadal, 2014; Muth et al., 2015; Grüner et al., 2019). The influence of the recognition effort in part 1 on part 2’s ratings is therefore of interest to us, and we will approach these analyses exploratively due to limited prior research.

In conclusion, our study aspires to shed light on the multifaceted process of Gestalt recognition by examining the dynamics of fast and slow perceptual processes and the timing of Aha. Additionally, our study aims to unravel the potentials and limitations of pupillometry in art research to enhance our understanding of cognitive and emotional responses evoked by visual art. Through these investigations, we hope to provide valuable insight into the complex mechanisms underpinning our appreciation of visual art and gain a deeper understanding of using pupillometry in art research.

### 1.1 Pupillometry as a potentially salient indicator for Gestalt recognition and Aha

Pupillometry—the analysis of pupil responses—provides a compelling method to detect sudden shifts in cognitive or affective states (Beatty and Lucero-Wagoner, 2000; Jepma and Nieuwenhuis, 2011; Laeng et al., 2012; Mathôt, 2018).^1^ It has been employed in various fields of study, such as working memory (Kahneman and Beatty, 1966), Stroop color-naming (Laeng et al., 2011), and figure-ground recognition tasks (Villani et al., 2015). Notably, pupil dilations have been linked with target detection during rapid serial presentation (Privitera et al., 2010), perceptual selection predicting subsequent stability in perceptual rivalry (Einhauser et al., 2008), and detection during subliminal repeating presentations (Laeng et al., 2012). However, pupil dilations also appear due to emotions (Hess, 1965; Bernick et al., 1971; Aboyoun and Dabbs, 1998), mainly associated with arousal and appeal of challenge (Hess and Polt, 1960; Muth et al., 2015), and pleasure in fluency (Kuchinke et al., 2009; Elschner et al., 2018). These findings suggest pupillometry holding promise as a yet underexplored method in art research for detecting recognition-related Aha moments and the presence of emotions.

Support for this approach is found not only in empirical data but also in neurology. Psychological (not light-induced) pupil responses are primarily modulated by the noradrenergic system in the locus coeruleus (Aston-Jones and Cohen, 2005; Gilzenrat et al., 2010; Jefferies and Di Lollo, 2019; Kret and Sjak-Shie, 2019). The locus coeruleus is a brain area associated with novelty perception and reward (Libby et al., 1973; Hervé-Minvielle and Sara, 1995). The fact that the locus coeruleus is involved in coordinating pupil reaction and pulling the strings during ongoing action/thought, focusing attention, and engaging in the notification of cognitive event boundaries, delivers a neurophysiological premise for using pupillometry as a key measure for notifying sudden moments.

To date, pupillometry has seen limited application in art recognition studies. Notable, two studies (i.e., Kuchinke et al., 2009; Elschner et al., 2018) leveraged pupil dilations to study fluency effects. In these studies, pupil responses were measured around the time of response, assumed to represent aesthetic emotions. While these studies provided valuable insights, they yielded contradictory results and only partially supported the idea of fluency-induced emotional response. In both studies, pupil responses were measured only around the time of response and were assumed to represent aesthetic emotions. Both studies asked participants to state with a key press when they recognized the artworks’ content—response times measured the processing fluency. Both studies found longer response times with increasing abstractness (reducing levels of accessibility). Kuchinke et al. (2009) focused on aesthetic emotions around the moment when participants recognized any figure in cubist paintings. In accordance with the hedonic fluency model, they found peak dilations just before stated recognition, which were larger with high-fluent artworks and positively correlated with preference ratings. Elschner et al. (2018), in a follow-up study, added expressionist abstract images. They found stronger dilations with decreasing levels of abstractness. The effect was stronger for cubist than expressionist art, although the latter was more liked. Hence, despite their effort to expand the design (additional styles; randomized instead of blocked style design), they did not find a fluency-induced emotional response reflected by dilations. It should be noted that art judgments were taken by participants who did not conduct the pupillometry experiment.

However, both studies detected peaks of pupil dilations at the moment of stated recognition. Given the study design that participants should recognize the content, maximum dilations may be interpreted as a physiological marker for Aha. As such, these studies can be reinterpreted as measures of Gestalt recognition, where pupil responses represented more a marker for Aha moment. However, the contradictory results could also have been grounded in the intermix of task and free viewing with no time limitation, yet participants should recognize the content of the artworks. In addition, both studies recorded pupil data short before the response and did not investigate the whole trial period. We address this limitation and explore not only average pupil dilation but also the time of the maximum pupil dilation covering both analyses of the whole trial period.

Building on this work, our study explores Gestalt recognition in abstract art as an Aha event in part 1 and as an affective event in part 2, measured by pupil dilations (time of maximum dilation and average dilation during the entire trial period) and potential associations with artwork judgments. We aim to delve deeper into this phenomenon, exploring not only the recognition process but also the strategies that participants might employ, from fluent, easy strategies to more meticulous and accurate ways of interaction. Note that we do not state that these working processes can be seen as being either perceptual, cognitive, or affective in quality. Instead, it is an interplay of all qualities. However, in part 1, we intend to focus the participants on the recognition task, and in part 2, we intend to give space for emotional response in a no-task condition and re-evaluation of their performance.

## 2 The present study

**Figure 1:**
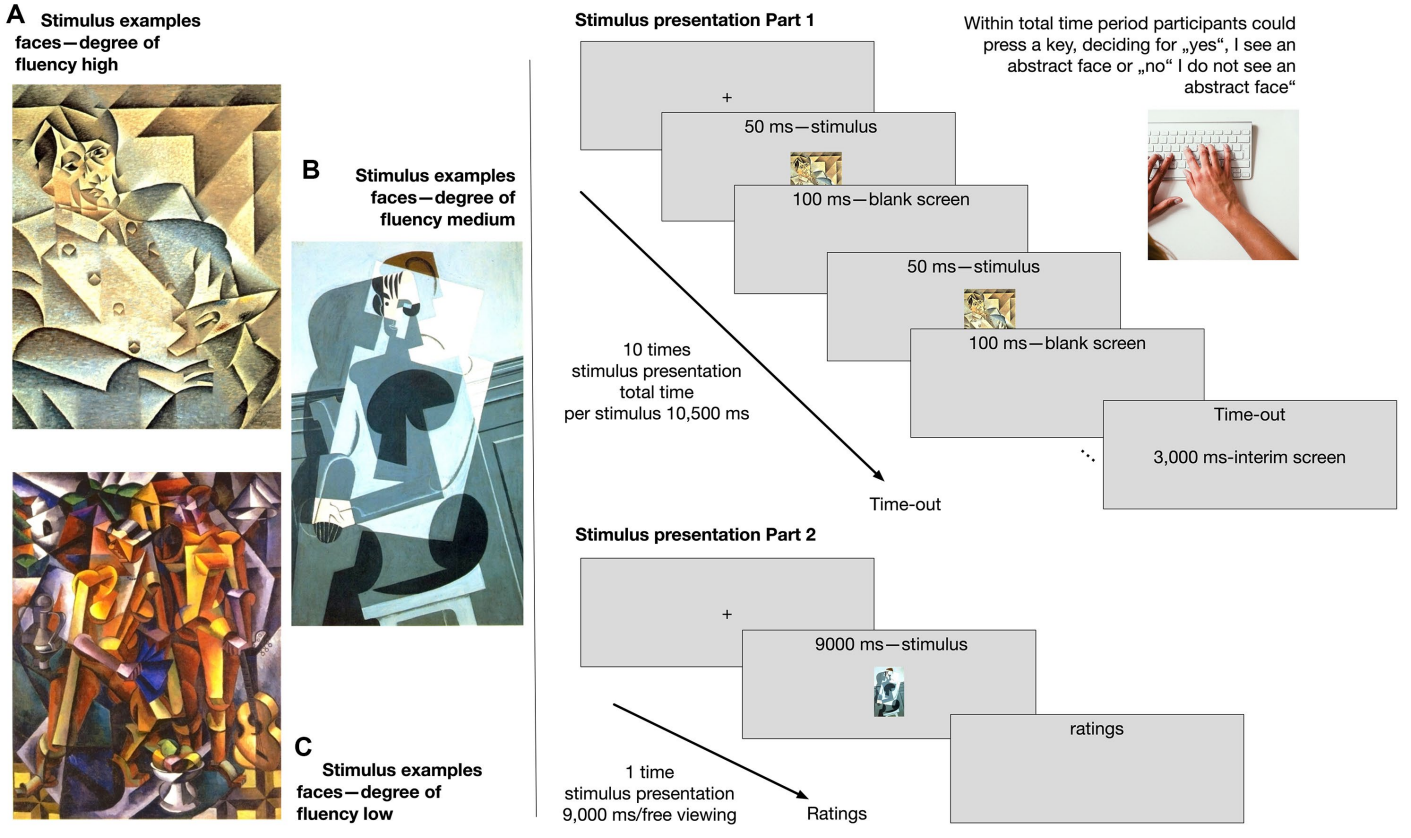
Left: Examples for stimulus content with faces differing in degree of fluency: high, medium, and low (see for the full list of artworks used Supplementary Table S1). Right: Stimulus presentation: in part 1 (right top), each artwork was presented 10 times for 50 ms in a row with 100 ms interim blank screens; in total, 10,500 ms. In the stimulus presentation in part 2 (right bottom), each artwork was presented one time for 9,000 ms. Afterward, the participants were asked to give their ratings on all six scales. Shown works are in the public domain in their country of origin and other countries and areas where the copyright term is the author’s life plus 70 years or fewer. These works are in the public domain in the United States because it was published (or registered with the U.S. Copyright Office) before 1 January 1926 (for image search and copyrights: https://commons.wikimedia.org/): **(A)** *Portrait of Pablo Picasso*. **(B)** *Portrait de Madame Josette Gris*. **(C)** *Composition with Figures*.

Our study, comprising two parts, seeks to understand Aha moments through Gestalt recognition in the context of visual cubist artworks. In part 1, we created a paradigm utilizing low-level artwork features to manipulate accessibility, varying from high to low fluency (see Figure 1). We incorporated an effort-taking context that required quick and accurate recognition of abstract faces in the artwork. This section involved a yes/no face-recognition task using short repeating stimulus presentations. Additionally, we instituted a public-private paradigm to manipulate the level of effort exerted during the task, emphasizing the goal of achieving Aha moments.

Depending on participants’ response times, we anticipate two possible outcomes. Short response times would support theories of perceptual fluency considering recognition and should correspond to higher liking ratings in part 2. This hypothesis suggests that recognizable faces in high-fluent artworks would be detected fastest. Conversely, if participants prioritize accuracy over speed, indicating longer response times for Aha experiences, it might suggest a blend of perceptual and top-down controlled mechanisms at play. We expect to observe the latter, particularly for high fluent and face stimuli, and aligning of timing of maximum pupil dilation with behavioral response.

In part 2, we examine pupil responses over an extended viewing period, exploring the average dilation and timing of maximum dilation. We also collected participant ratings post-stimulus presentation to ascertain potential correlations between pupil responses, arousal, image clarity, liking, complexity, comprehension, and emotional valence. Finally, we investigated the influence of confidence in one’s correctness on artwork ratings in part 2, examining the effects of correct and missed recognition made in part 1. This approach offers a holistic view of the interplay between cognition, emotion, and physiological responses in the context of art perception and recognition.

## 3 Materials and methods

### 3.1 Participants

The final sample included 84 participants (*M*_age_ = 21.27, SD = 2.16; 57.1% female) from an original sample of 106 mainly psychology students from the University of Vienna. From the original sample, 22 participants were excluded due to technical issues (*n* = 16) or input recording (*n* = 6). Another *n* = 14 participants had to be excluded only for the pupil analysis (final eye-tracking data analysis *N* = 70), as specific raw data files for several participants were not saved. Course credit was given for participation. No participant reported an academic background in fine arts, art history, or other related disciplines dealing with art. A pre-online questionnaire ensured that none of the participants was color-blind or had more than 1.2 diopters of visual impairment. All participants signed informed consent, and the ethics committee of the University of Vienna approved the study.

### 3.2 Apparatus

Pupil measures were recorded with a video-based Eyelink 1,000 desktop-mounted eye-tracker (SR Research Ltd., Mississauga, Ontario, Canada). An infrared-sensitive camera provided pupillometry at a sampling rate of 1,000 Hz. The experiment was controlled by Experiment Builder Software Version 1.10.1630 (SR Research Ltd., Mississauga, Ontario, Canada) on a Windows PC. EyeLink 1,000 has implemented two pupil tracking algorithms: centroid and ellipse fitting. We used the centroid mode, tracking the center of the threshold pupil using a center of the mass algorithm.

### 3.3 Stimuli

The stimuli were the 39 cubist paintings from Kuchinke et al. (2009), differing in their degree of accessibility (see Figure 1, left, see Supplementary Table S1, for a full list of artworks). The accessibility level was pre-rated in their study. We used three levels of accessibility: high, medium, and low. These were further divided into stimulus contents (with visible abstract/figurative faces or landscapes). Of the 39 artworks, 30 paintings were used as stimuli in the present study (10 images for high-fluent/50% faces, 11 for medium-fluent /45% faces—9 for low-fluent/55% faces), and nine paintings were used for warm-up trials. The stimuli subtended at maximum a vertical visual angle of 17.19° and a horizontal visual angle of 15.28° (based on Kuchinke et al., 2009). The stimuli were presented on an LCD monitor (SyncMaster 2443BW, Samsung) with a resolution of 2,400 by 1,920 pixels and a screen refresh rate of 60 Hz. The images were edited to mean luminance (both ps > 0.180; luminance high-fluent = 156.46 cd/mm^2^, high-fluent = 159.55 cd/mm^2^, low-fluent = 162.40 cd/mm^2^) to reduce the influence of luminance (Loewenfeld, 1999; Kuchinke et al., 2009).

### 3.4 Calibration measurements and luminance issues

Participants were tested in a medium-lit room, sitting 60 cm from a monitor with their heads on a chin rest. The dominant eye was determined, and its movements were calibrated with EyeLink 1,000. To avoid undesirable pupil reactions due to luminance differences, a single image was displayed at the screen’s center, optimized for accurate viewing. The calibration quality was maintained within the pupil box preset of EyeLink 1,000, with camera setup and calibration repeated as needed to avoid corneal reflection loss. Two calibrations per participant were performed before the experiment. Pupil measurements were taken monocularly (dominant eye) during a fixation, with noise levels limited to 0.2% of the pupil diameter. Pupil size was reported in arbitrary units. This corresponds to a resolution of 0.01 mm for a 5 mm pupil. Pupil size was not taken in commonly used micrometers, but in units of the EyeLink 1,000 system default. Pupil size reported by EyeLink 1,000 is an integer number in arbitrary units (= “au,” system-typical pupil diameter measures ranged from 400 to 16,000 units). Participants were asked to minimize blinking and avoid looking around during trials.

Potential pupil responses due to initial light reflexes and size fluctuations from the blank screens between image presentations were anticipated. Full luminance control was not possible in part 1, and we opted not to use scrambled image versions between screens to avoid disrupting recognition processes. However, prior research has successfully used pupillometry in similar repeating and subliminal stimulus presentations (Einhauser et al., 2008; Ionescu, 2016). Unlike prior art studies (Kuchinke et al., 2009; Elschner et al., 2018), we analyzed the entire trial duration in both parts. In part 2, the 9,000 ms static image viewing period, we have posed no luminance issues.

### 3.5 Procedure

All instructions were given in written form on the computer screen. Participants knew that the experiment had two parts (part 1: face-recognition task; part 2: viewing and rating task).

In the task of part 1, participants were asked to press either a yes-key or a no-key (on a normal keyboard, left/right key-assignment counterbalanced) when they recognized ‘yes, there is a face or faces’ or ‘no, there is no face or faces’ in the cubist paintings presented. Nine warm-up rounds ensured that participants understood the task. To start the trial, participants had to fixate on a center cross for at least 220 ms. Afterward, an artwork was displayed a maximum number of 10 times in rapid succession for 50 ms each time with an interval of 100 ms in between per flashes (see Figure 1, top right). In total, participants had a maximum of 10,500 ms time to make their decisions. If the participant did not press any key in time, the time-out was noted, and a new trial with a new artwork started. After each trial, a 3,000 ms blank screen was presented, allowing a short pause, after which participants had to fixate on the fixation cross again, followed by a new trial. In total, the participants had 30 trials/artworks to give their yes or no response.

In part 2, participants were told that they were shown the same artworks again for a fixed-time duration of 9,000 ms each (duration was based on the study results of Kuchinke et al., 2009, where average recognition time was 9,000 ms). After 9,000 ms, the image disappeared from the screen. Participants gave their ratings in arousal, emotional valence, liking, complexity, comprehension, and clearness using a 7-point Likert-type scale, ranging from 1 (not at all or negative) to 7 (very much or positive). Regarding valence and arousal, we explicitly asked the participants to focus on their subjective felt elicited response, meaning what the artwork elicited in them.

#### 3.5.1 Variation of effort motivation

The between-subjects factor effort motivation varied only in part 1 (i.e., face-recognition task). Participants were assigned counterbalanced to either a public (*n* = 42) or a private condition (*n* = 42). To manipulate the participants’ motivation in making an effort to focus on the Gestalt recognition task, in the public condition, participants were (wrongly) informed that their performance would be ranked in a high-score list and discussed in the next research seminar for further face-recognition evaluation reasons (for similar manipulation, see Van Honk et al., 2016). In the private condition, participants were assured in the instruction that their performance and effort were anonymous and private.

### 3.6 Baseline correction, preprocessing, and usage of statistical analysis

Pupil data were analyzed in Python using custom code for preprocessing and the scipy, statsmodels (Seabold and Perktold, 2010), and the pingouin (Vallat, 2018) libraries for statistics. Blinks were detected as missing values in the signal. We removed pupil data around the blinks and replaced it with nan-s (no interpolation) in a time window of 20 ms before and after each blink. Additionally, ±20 ms pupil data were removed around sudden sharp changes in the signal using a threshold of 3.5 SD’s, as these are usually an indication of ocular artifacts (Mathôt et al., 2018).

To account for different baseline diameters in pupil dilation, a baseline correction was conducted. We calculated baselines for each individual trial by using the mean pupil size during participants’ fixation on the cross, which was shown before each trial. We followed the criteria suggested by Mathôt et al. (2018) for data exclusion, although no participants from the final sample reported in the results had to be excluded.

Additionally, we applied multivariate analysis and linear mixed models (LMMs). Stimulus content (faces and landscapes) and fluency (high, medium, and low) varied as within-subject factors and effort motivation (public versus private) as a between-subject factor (Baayen et al., 2008; Hox et al., 2010; Bosker and Snijders, 2011; Gałecki and Burzykowski, 2013; Brieber et al., 2014).

## 4 Results

The results are presented as follows: (1) first, we describe the results of the behavioral outcomes in part 1 (response times for each main factor), and then, we present (2) the results of the pupil analysis along two aspects: the average pupil dilation and the time of the maximum pupil dilation within each trial for both part 1 and part 2. (3) For part 2, we describe the rating results, which we again complement with pupil data. Finally, we show (4) explorative analyses connecting both parts.

### 4.1 Behavioral analysis of part 1

Descriptive analysis is shown in Supplementary Table S2. We assessed the relation of the response time between the various conditions by using LMMs. For this analysis, we included only the results from the high- and low-fluent artworks since there were no differences in means between the medium- and low-fluent conditions (including the medium condition was also not shown to change the following main results). We used the response time for each stimulus as the dependent variable. The independent variables were performance outcome (dummy-coded) with the correct response (hit) as a baseline against both error types, false-alarm (false assessment) and misses (missed target), effort motivation (dummy-coded) with private as the baseline against the public, stimulus content (dummy-coded) with faces as the baseline against landscape, and the degree of fluency (dummy-coded) with low fluent as the baseline against high fluent. We further included the interaction between the degree of fluency and performance outcome. Finally, we estimated the intercept as a random coefficient (the intercept could vary between participants). Time-outs were excluded from the analysis.

We found significant main effects for the degree of fluency and stimulus content. Participants had 70.47 ms shorter response times for landscape artworks than faces. The main effect of fluency resulted in longer response times (by 60.13 ms) in the high-fluent than the low-fluent condition. Misses and false alarms did not differ significantly from correct answers in response times, and there was also no difference in response times due to effort motivation. To summarize, participants had longer response times for stimuli with faces and high-fluent artworks (see Table 1).

**Table 1:** Part 1, fixed effects in the LMM predicting response times; baselines are represented by stimulus content—face(s), degree of fluency—low fluent as it represented the most difficult way to solve the artwork, and performance-outcome—correct.

		95% CI			
Fixed effects	Estimate	Lower	Upper	*t*-value	Pr(>|*t*|)
Intercept	506.62	472.78	540.48	29.36	< 0.01
High fluent	59.04	30.56	87.52	4.06	< 0.01
False alarm	−31.51	−121.34	58.32	0.69	0.492
Miss	−31.39	−78.97	16.19	1.29	0.20
Effort-motivation—public	−14.03	−48.15	20.10	0.81	0.42
Stimulus content—landscape	−95.56	−123.48	−67.65	6.71	< 0.01
Interaction between High fluent—false-alarm	−14.16	−131.91	103.59	0.24	0.81
Interaction between High fluent—miss	−111.77	−282.05	58.50	1.29	0.20

### 4.2 Pupil analysis

We focused on two main aspects for the analysis of the pupil data: (1) the average pupil dilation in each trial and (2) the time of maximum pupil dilation within each trial. Since the trials in part 1 had different lengths, the time of the maximum was calculated relative to the total length of each trial.

#### 4.2.1 Mixed model results for average pupil dilations—part 1

Our first analysis looked at the average pupil dilation within the whole trial period. We found that neither of our main manipulations, *stimulus content* (*p* = 0.37, Table 2) and *accessibility* (*p* = 0.60), influenced average baseline subtracted pupil size. There was a significant negative association with the trial number (*p* = 0.04), suggesting that the average pupil change from baseline was getting smaller across trials. Adding *performance outcome* as a predictor did not change the main results as mean pupil dilation for correct and incorrect responses were not different (*p* = 0.68, Supplementary Table S3). Finally, adding *effort motivation* as a predictor also had no significant influence on average dilation (*p* = 0.07, Supplementary Table S3).

**Table 2:** Part 1 and part 2, results of multi-mixed methods for baseline-corrected average pupil size.

Fixed effects	Estimate	SE	Lower	Upper	*z*-value	Pr(>|z|)
*PART 1*	95% CI					
Intercept	−80.40	10.66	−101.30	−59.40	−7.54	<0.001
Accessibility	1.97	3.78	−5.45	9.39	0.52	0.603
Stimulus content	5.51	5.97	−6.20	17.21	0.92	0.356
Trial	−0.71	0.34	−1.39	−0.04	−2.08	0.038
*PART 2*			95% CI			
Intercept	66.57	23.41	20.69	112.45	2.84	0.004
Accessibility	−25.96	4.56	−34.90	−17.01	−5.69	<0.0001
Stimulus content	33.38	7.12	19.25	47.52	4.63	<0.0001
Trial	−1.52	0.41	−2.33	−0.71	−3.66	<0.0001

The dependent variable was average pupil dilation. Independent variables were accessibility, stimulus content, and trail number.

#### 4.2.2 Mixed model results for the time of maximum pupil dilation—part 1

The analysis of the time of maximum pupil dilation showed a different pattern. There was a significant negative association with *accessibility* (*p* < 0.001, Table 3), showing that low fluency predicted earlier maximum dilation. At the same time, the predictor *stimulus content* had a positive influence (*p* < 0.001, Table 3), showing that face stimuli had later maximum dilation than landscapes.

**Table 3:** Part 1 and part 2, results of multi-mixed methods for maximum pupil size.

Fixed effects	Estimate	SE	Lower	Upper	*z*-value	Pr(>|z|)
*PART 1*	95% CI					
Intercept	0.33	0.30	0.27	0.39	11.12	<0.001
Accessibility	−0.05	0.01	−0.07	−0.03	−5.14	<0.001
Stimulus content	0.09	0.02	0.06	0.12	5.94	<0.001
Trial	0.00	0.00	0.00	0.00	4.23	<0.001
*PART 2*			95% CI			
Intercept	0.15	0.02	0.11	0.19	7.24	<0.0001
Accessibility	0.00	0.00	−0.00	0.01	0.89	0.376
Stimulus content	−0.01	0.01	−0.02	0.00	−1.46	0.145
Trial	−0.00	0.00	−0.00	−0.00	−2.53	0.011

The dependent variable was the time of the maximum pupil dilation. Independent variables were accessibility, stimulus content, and trail number.

Adding *performance outcome* as a predictor also showed a significant influence (*p* = 0.001, Supplementary Table S4) in predicting later maximum pupil dilation on correct response trials. Finally, *effort motivation* as a predictor did not show significance (*p* = 0.67, Supplementary Table S4). Adding both latter predictors did not change the results of the three other predictors, which remained very similar.

#### 4.2.3 Mixed model results for average pupil dilations—part 2

The results in part 2 showed a very different pattern from part 1. Here, all our main predictors were significant in the prediction of baseline-corrected average pupil dilation (Table 2). *Accessibility* had a negative influence (*p* < 0.0001), showing that high-fluent artworks led to larger pupil dilation. Additionally, *stimulus content* had a positive effect (*p* < 0.0001), showing that dilation was larger for artworks with faces than landscapes. Finally, as in part 1, there was a significant negative effect of trial number (*p* < 0.0001), stemming from a smaller pupil dilation over time.

#### 4.2.4 Mixed model results for the time of maximum pupil dilation—part 2

As opposed to the analysis of average pupil dilation, the analysis of the temporal position of maximum pupil dilation uncovered no significant effects (Table 3) in part 2 for our main predictors. The control trial number was significant (*p* = 0.011), but unlike in part 1, neither *accessibility* (*p* = 0.376) nor faces (*p = *0.145) were significant predictors.

### 4.3 Analysis of artwork ratings—part 2

Descriptive statistics of the ratings, differentiated by *stimulus content* and *accessibility*, are reported in Table 4. Supplementary Figure S1 shows a correlation heatmap of all ratings.

**Table 4:** Part 2, means and standard deviation of ratings (7-point Likert-type scale, ranging from 1 = not at all or negative valence to 7 = very much or positive valence) between the different stimuli presentations and degree of fluency.

Stimulus-condition	Accessibility	Complexity	Comprehension	Valence	Arousal	Clearness	Liking
		*M* (SD)	*M* (SD)	*M* (SD)	*M* (SD)	*M* (SD)	*M* (SD)
Faces	High	4.08 (0.95)	4.58 (0.85)	3.41 (0.76)	4.45 (0.75)	6.65 (0.47)	3.88 (1.01)
	Medium	4.55 (0.84)	3.46 (0.98)	3.82 (0.55)	4.47 (0.72)	4.70 (0.82)	3.80 (0.88)
	Low	5.16 (0.72)	2.73 (0.93)	3.93 (0.61)	4.37 (0.78)	3.55 (1.08)	3.71 (0.90)
Landscapes	High	3.89 (0.81)	4.30 (1.00)	4.59 (0.79)	3.15 (0.83)	1.43 (0.54)	4.44 (0.87)
	Medium	4.08 (0.78)	3.94 (1.04)	4.57 (0.71)	3.40 (0.83)	1.25 (0.38)	4.37 (0.87)
	Low	5.01 (0.75)	3.15 (0.93)	3.86 (0.83)	4.18 (0.72)	1.50 (0.57)	4.09 (0.99)

**Figure 2:**
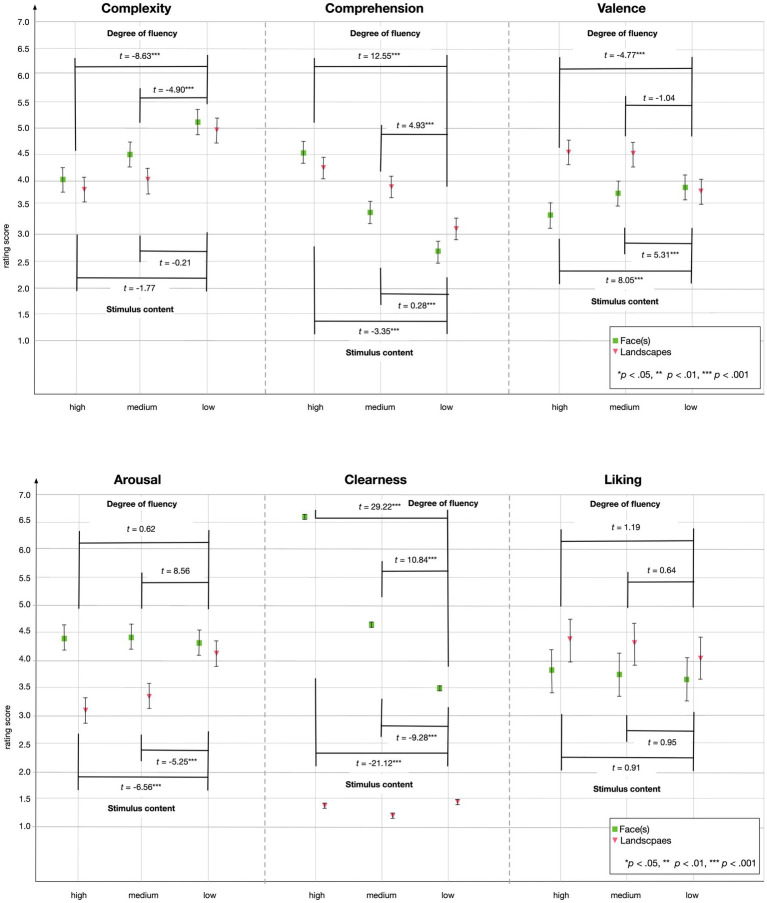
Linear mixed models for each rating scale as dependent variable. Stimulus content (set ‘face(s)’ as the baseline) and accessibility (set ‘low-fluent’ as the baseline) were set as the independent variables and estimated the intercept as a random coefficient (the intercept could vary between participants).

We used a series of LMMs for each rating scale with *stimulus content* (set ‘face(s)’ as the baseline) and *accessibility* (set ‘low-fluent’ as the baseline) as the independent variables, and we estimated the intercept as a random coefficient (the intercept could vary between participants). The structure of the fixed effects and the random effect were identical across all rating scales (Figure 2).

#### 4.3.1 Pupil responses and art ratings—part 2

**Figure 3:**
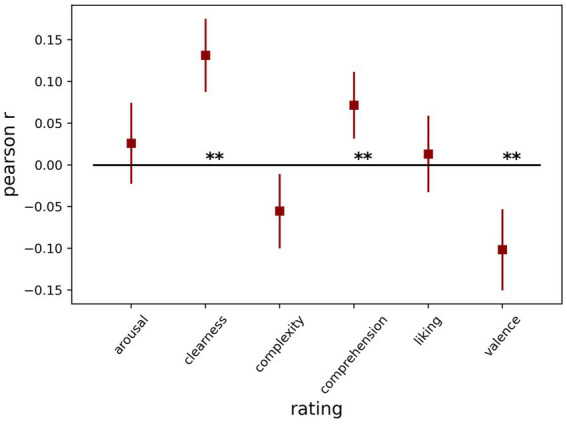
Average correlation with pupil size for each of the ratings.

In part 2, we expected that the ratings could be related to the average pupil dilation. First, we looked at the Pearson correlation between each of the ratings and dilation across trials for each participant separately. Next, we tested the *r* values against zero across participants in a one-sample *t*-test for each rating type. We found that clearness had a strong positive (*t* (69) = 5.89 *p* < 0.001), while valence had a strong negative association (*t* (69) = 4.09 *p* < 0.001) considering pupil size, meaning the more positive the image was, the more the pupil dilated. Additionally, comprehension had a smaller but significant (*t* (69) = 3.52, *p* < 0.001) positive association with pupil size. The other three ratings did not show a significant relationship with pupil size [arousal: *t* (69) = 1.05, *p* = 0.299; complexity: *t* (69) = 2.44, *p* = 0.017; liking: *t* (69) = 0.56, *p* = 0.574], after correcting for multiple comparisons. Figure 3 shows the average correlation with pupil size for each of the ratings.

Next, we re-analyzed the same data with LMMs, including all six art ratings as predictors, and baseline-corrected pupil dilation as the outcome (Table 5). Additionally, we included the trial number as a control variable and a random intercept for each participant. We confirmed the strong association of negative valence (*p* < 0.001) and clearness (*p* < 0.001), while comprehension (*p* = 0.484) and arousal (*p* = 0.312) were not significant. Liking (*p* = 0.048) and complexity (*p* = 0.034) were associated with pupil size only before the Bonferroni correction for multiple comparisons. The results differ from those using the Pearson correlation in the combined model as some ratings are slightly correlated (see Supplementary Figure S1).

**Table 5:** Part 2, Linear mixed models for artwork ratings and baseline-corrected average pupil size.

			95% CI			
Fixed effects	Estimate	SE	Lower	Upper	*z*-value	Pr(>|z|)
Intercept	78.53	30.12	19.50	137.57	2.61	0.009
Arousal	−2.87	2.84	−8.44	2.69	−1.01	0.312
Clearness	6.76	1.63	3.56	9.95	4.14	<0.0001
Complexity	−6.16	2.90	−11.84	−0.48	−2.12	0.034
Comprehension	1.97	2.82	−3.55	7.49	0.70	0.484
Liking	6.02	3.04	0.06	11.98	1.98	0.048
Valence	−14.86	3.45	−21.62	−8.09	−4.31	<0.0001
Trial	−1.51	0.42	−2.33	−0.69	−3.60	<0.0001

### 4.4 Explorative results combining parts 1 and 2

#### 4.4.1 Analysis of performance outcome in part 1 on ratings in part 2

To analyze the effects of performance outcome in part 1 on ratings in part 2, we took as independent variables the answer type correct/hit, false alarm, and miss, and we calculated LMMs for all ratings as dependent variables. Again, correct answers were taken as the baseline and the intercept as a random coefficient (the intercept could vary between participants).

We found that false alarms (meaning that participants responded that they saw faces although it was a landscape) were associated with lower ratings for clearness [−1.56 points on average, *t* = 6.89, *p* < 0.01, 95% CI (−2.00, −1.10)], higher liking [0.42 points on average, *t* = 2.89, *p* < 0.01, 95% CI (0.14, 0.71)], and more positive valence [0.40 points on average, *t* = 2.16, *p* < 0.01, 95% CI (0.15, 0.65)], compared to correct answers. However, there were no significant differences in arousal, comprehension, or complexity when participants made the false-alarm errors. When participants misidentified an image with faces as a landscape (miss), results showed that misses were associated with higher ratings of arousal [0.44 points on average (oa), *t* = 5.09, *p* < 0.01, 95% CI (022, 0.61)] and complexity [0.51 points oa, *t* = 6.01, *p* < 0.01, 95% CI (0.34, 0.68)]. Finally, liking [−0.54 points oa, *t* = 5.81, *p* < 0.01, 95% CI (−0.73, −0.36)], clearness [−0.43 points oa, *t* = 2.97, *p* < 0.01, 95% CI (−0.72, −0,15)], and positive valence [−0.27 points oa, *t* = 3.39, *p* < 0.01, 05% CI (−0.43, −0.12)] were all lower in turn. For the full report, see Supplementary Table S5.

#### 4.4.2 Combined analysis of average pupil dilation—part 1 and part 2

In general, the average baseline-corrected pupil dilation was −84.24 ± 49.06 au. in part 1 and − 33.25 ± 96.24 au. in part 2, showing that the average dilation was smaller in part 1 (*t*_69_ = 4.3, *p* < 0.0001, *d* = 0.66, Supplementary Figure S2a, see also Supplementary Figure S2b, where the effect of the flashing stimulus presentation resulting in wave-like patters is visible in the pupil data in part 1).

**Figure 4:**
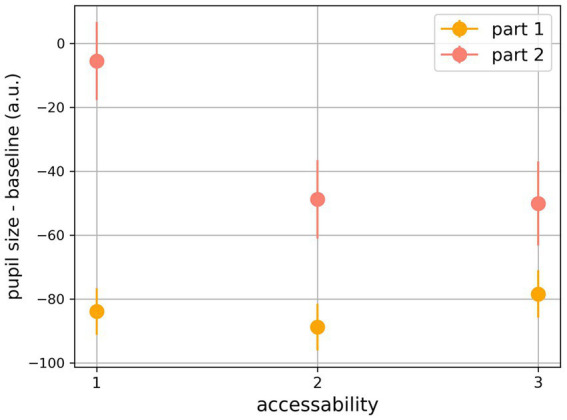
Combined analysis of average pupil dilation for the two experimental parts considering accessibility (baseline-corrected and preprocessed pupil data).

In the combined analysis of average pupil dilation for the two parts and *accessibility*, we used a two-way repeated-measures ANOVA (see Figure 4). We found that average baseline-corrected pupil dilation was significantly higher in part 2 (*F* (1,69) = 17.18, *p* < 0.001). There was also a significant effect of *accessibility* (*F* (2,138) = 10.04, *p* < 0.001); this effect of accessibility was more pronounced in part 2, resulting in an interaction between *accessibility* and the experimental parts (*F* (2,138) = 10.93, *p* < 0.001). See results for both, *accessibility* and *stimulus content*, considering raw data in the Supplementary Figures S3a,b.

**Figure 5:**
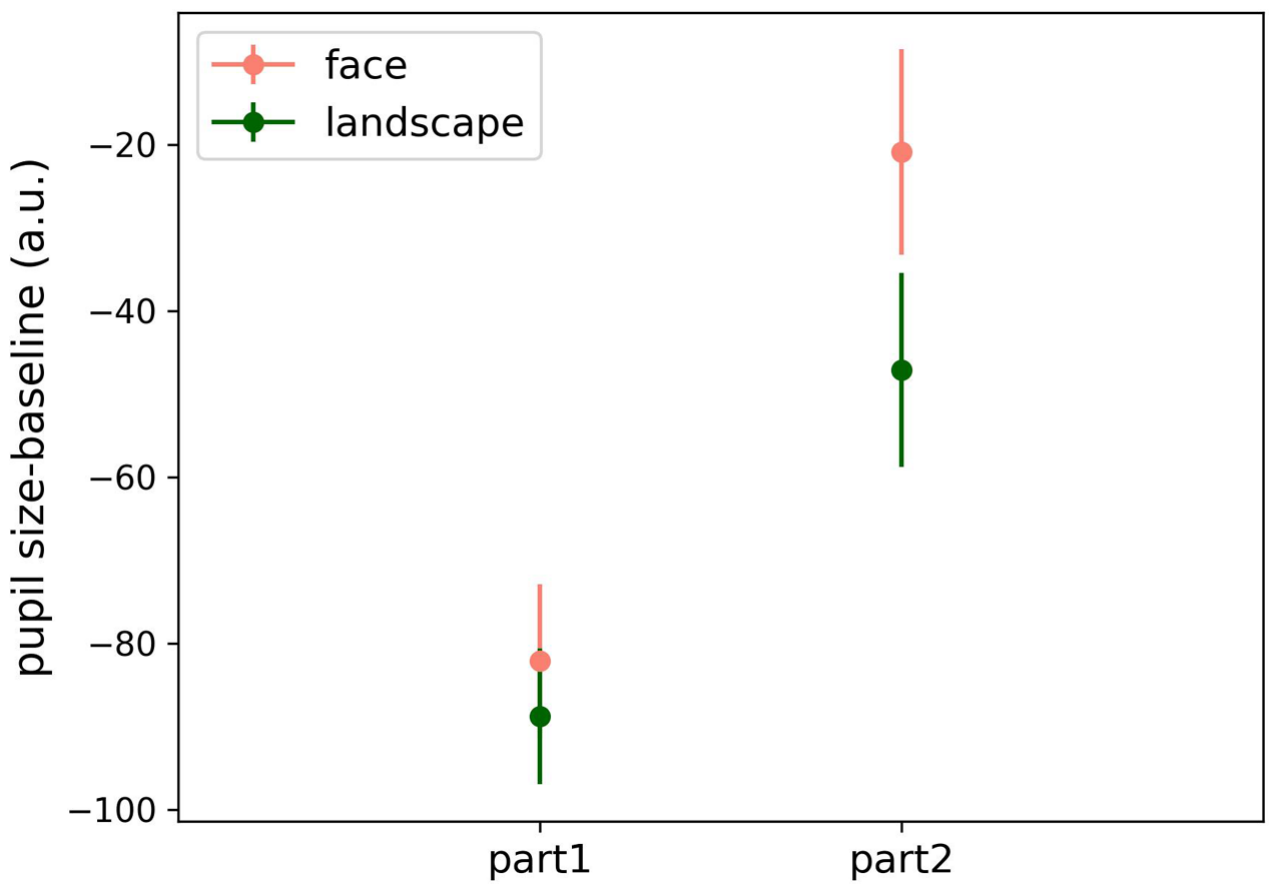
Combined analysis of average pupil dilation for the two experimental parts considering stimulus content (baseline-corrected and preprocessed pupil data).

The combined analysis of the experimental parts and *stimulus content* (see Figure 5) was also performed with a two-way repeated-measures ANOVA. We found that, in general, baseline subtracted pupil dilation for face stimuli was larger (*F* (1,69) = 9.659, *p* = 0.003), and there was also a significant effect of the experimental parts (*F* (1,69) = 16.35, *p* < 0.001), with a far larger pupil dilation in part 2. There was no significant interaction between the factors (*F* (1,69) = 3.01, *p* = 0.087).

#### 4.4.3 Combined analysis of time of maximum pupil dilation—part 1 and part 2

**Figure 6:**
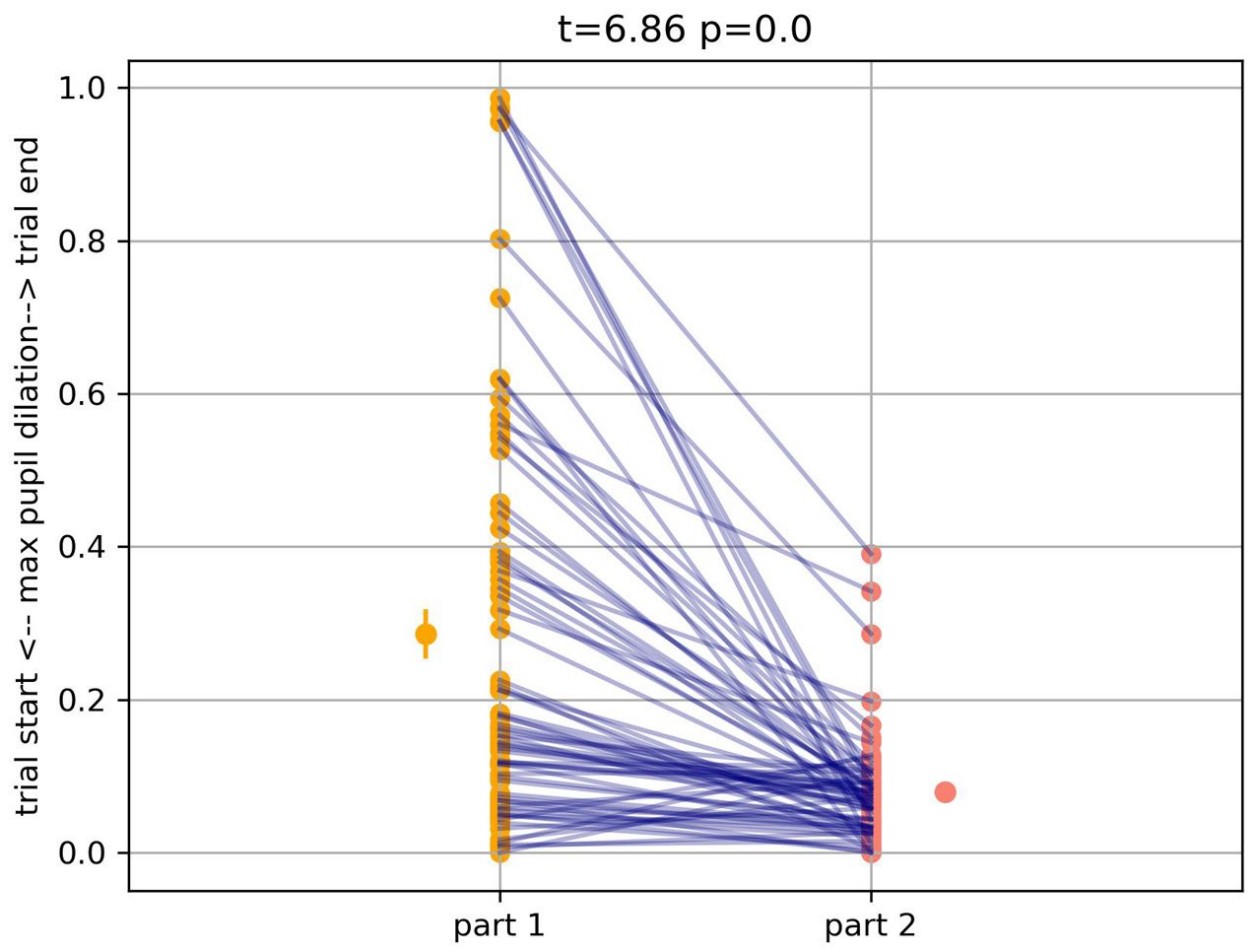
Time of the maximum pupil dilation, with later dilations in part 1 (*t*_69_ = 6.86, *p* < 0.0001, *d* = 1.04; 0.33 ± 0.16, units in proportion of trial), compared to part 2 (0.11 ± 0.08, units in proportion of trial).

Considering the time of the maximum pupil dilation, results show that the maximum pupil dilation was later in part 1 (*t*_69_ = 6.86, *p* < 0.0001, *d* = 1.04; 0.33 ± 0.16, units in proportion of trial), compared to part 2 (0.11 ± 0.08, units in proportion of trial, see Figure 6). Finally, when *accessibility* was included as a factor in a repeated measures ANOVA, we found that this measure was also influenced by *accessibility* (*F* (2,138) = 7.39, *p* > 0.001), with a strong effect of the experimental parts (*F* (1,69) = 17. 16, *p* < 0.001) and an interaction between the experimental parts and *accessibility* (*F* (2,138) = 10.27, *p* < 0.001). Notably, the effect of *accessibility* on dilation was significant, but negative in part 1 (Table 3).

**Figure 7:**
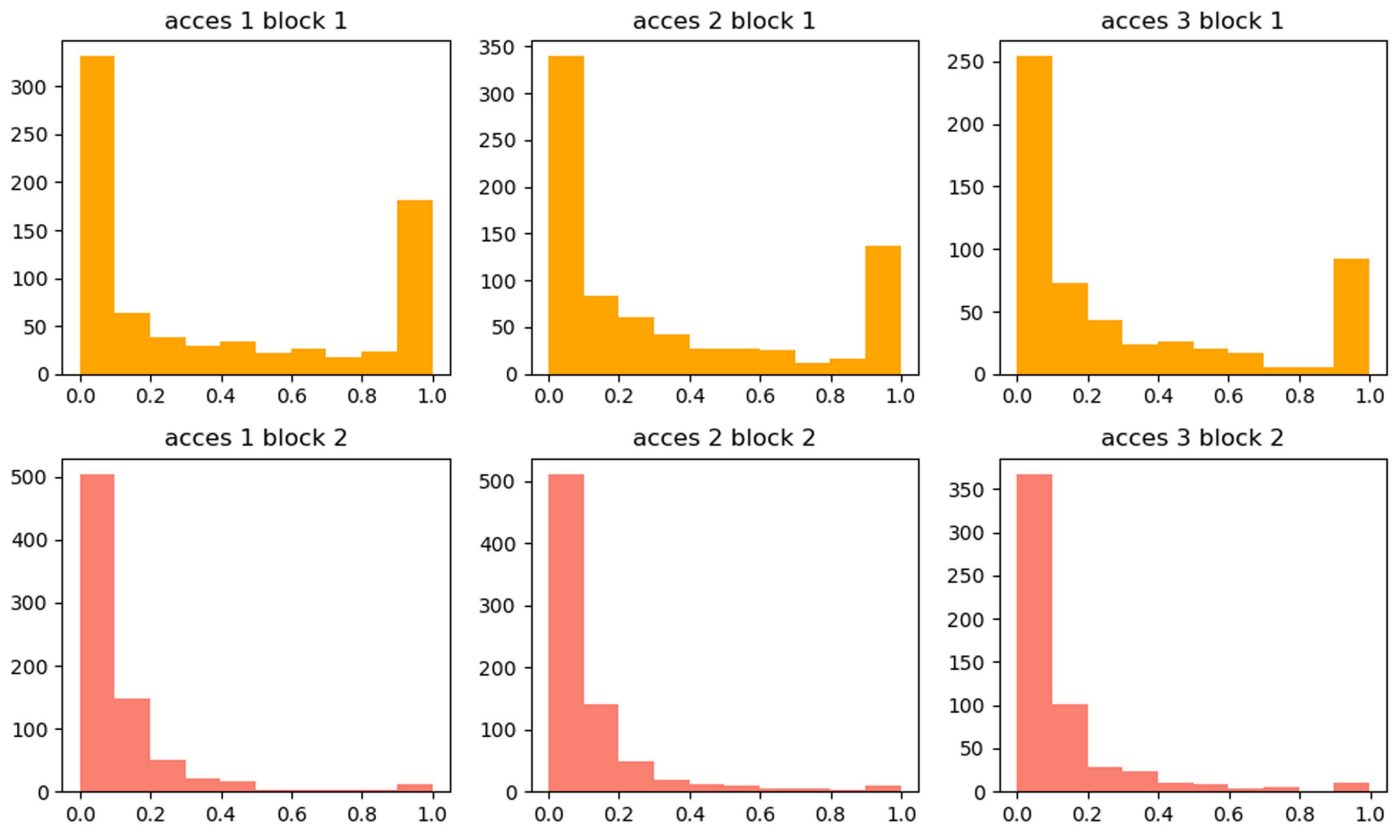
Distribution of trials with respect to the relative time of maximum pupil dilation. The *x*-axis shows the proportion of trial time, where the maximum pupil dilation was. The *y*-axis is the number of trials.

Most importantly, in contrast to prior studies (Kuchinke et al., 2009; Elschner et al., 2018), we analyzed the whole trial period for each trial and connected this to pupil dilations in both parts. Figure 7 shows the distribution of trials with respect to the relative time of maximum pupil dilation. Here, it shows that even though we found an association with our main predictors, *stimulus content* and *accessibility*, the maximum pupil dilation did not always coincide with the behavioral response. In part 1, we found an approximately bimodal distribution, with the maximum dilation mostly at the beginning or end of the trial. In part 2, the maximum dilation was mostly at the beginning.

## 5 Discussion

In studying the phenomenon of Gestalt perception, we enter a fascinating domain where our minds seamlessly form holistic concepts from noisy/fragmented sensory input (Wertheimer, 1923; Wagemans, 2013). This phenomenon is particularly captivating when this process involves an Aha moment, a sudden insight where abstract and first disconnected elements within a visual artwork converge into a meaningful, recognizable whole (Arnheim, 1954; Muth et al., 2013). This moment of recognition can vary from being immediate to delayed, where both encounters impact our experience with, our aesthetic judgment of, and our emotional response to art (Van de Cruys and Wagemans, 2011a,b; Reber, 2012; Muth and Carbon, 2013). While the recognition of Gestalt patterns is pivotal in experiencing these Aha moments, particularly in the context of cubist art, it forms only a part of the broader evaluative process. In cubist art, the recognition of Gestalt forms a foundation for evaluation, but this evaluation is more comprehensive, entailing the integration of these identified patterns with the entire artwork to elicit an overall emotional and, sometimes even an aesthetic, response. In our design, we emphasized the art-task instructions to focus participants on experiencing an Aha moment/recognition (part 1) or free-viewing and evaluating (part 2). This process aligns with the multi-stage models of art perception and evaluation (see, e.g., Leder et al., 2004; Leder and Nadal, 2014; for further reading, Pelowski et al., 2016, 2017), which highlight not only the cognitive recognition of art elements but also the emotional engagement with and evaluation of the artwork.

### 5.1 Behavioral results

In part 1, we analyzed response times based on the following three main factors: stimulus content, accessibility, and performance motivation manipulated by a private versus public design. Our results showed longer response times for highly fluent artworks and face stimuli, supporting our hypotheses. However, no significant findings were identified for the performance outcome. These findings contradict the hedonic fluency model (Belke et al., 2010; Reber, 2012; Jakesch et al., 2013; Elschner et al., 2018), instead favoring theories that suggest a delayed, meaningful, and accurate recognition process (Van de Cruys and Wagemans, 2011a,b; Van de Cruys, 2017).

The intriguing result of longer response times for recognizing faces challenges the conventional understanding that configural processing during face recognition is highly efficient, resulting in quick detection (Leder and Bruce, 2000; Sandford and Bindemann, 2020). It suggests that the task’s nature and our attitude when encountering art can influence the visual art interaction mechanism, requiring more cognitive effort and time to decipher highly fluent artworks and faces, when attention is paid to this effort (Aston-Jones and Cohen, 2005). Further analysis showed similar response times for landscapes and medium- to low-fluent face stimuli. This could indicate that participants either took their chances or adopted a superficially fast attitude that did not require deep attention.

We further would redefine the Aha moment from an instantaneous revelation to a potentially more “calculated slow” than “reflective fast” cognitive process. Our findings align with our hypothesis of prioritizing accurate interpretation and well-founded predictions. The observed delay in cognitive further shift suggests top-down control at a behavioral level, demanding further exploration in neuroscientific research (Van de Cruys et al., 2017; Van Geert and Wagemans, 2020).

Considering ratings, our results indicate a nuanced interplay between fluency and the emotion ratings valence and arousal. Valence ratings were more positive for low-fluent face stimuli but more negative for low-fluent landscapes. Landscapes showed more positive valence scores for high- and medium-fluent artworks, where face stimuli gained higher arousal ratings in general. Liking was indifferent to both stimulus content and accessibility in all categories. Clearness was particularly different for faces, with decreasing clearness ratings along with decreasing accessibility levels. Landscapes were rated as quite unclear in general. As these results do not show a clear association between preference, emotional valence, and comprehension considering content and accessibility, they hint at a potential influence of task and performance outcome. An analysis, which we conducted exploratively.

Our analyses reveal intriguing effects of performance outcomes of part 1 on artwork ratings in part 2. When participants falsely recognized faces in landscapes (false-alarm), they tended to rate the artwork as less clear, but they liked them more with more positive valence ratings than correct responses. No significant differences were observed in arousal, comprehension, or complexity ratings in these instances. Conversely, when participants failed to identify faces in the artwork (miss), the artwork was perceived as more arousing and complex; such errors were further associated with negative valence and lower ratings of liking, clearness, and comprehension.

One possible interpretation considering the art field is that false alarms are experienced as non-threatening, pleasurable, and positive as they reflect the artists’ skill in leaving ambiguity to a level where much can be seen, even though it is not there. On the contrary, not detecting a face, which is actually there, can be experienced as a failure in detecting the right meaning. These insights into how recognition errors influence judgment and affective responses to artwork could offer valuable contributions to the understanding of the cognitive and emotional landscapes of art appreciation. In addition, our results suggest that art appreciation is based on a more intricate interplay of cognitive state, task (or rather attitude), and affect, and not strictly on perceptual clarity or fluency (Seth, 2013, 2019). Future research could explore state attitudes of people interacting with art and measure their varying experiences. This is relevant considering factors such as social context (Fingerhut, 2020) in a crowded museum or perceived ability to decipher abstract patterns, which could influence the level of in-depth processing and attention.

### 5.2 Pupillometry

Our study design aimed to integrate an implicit measure for notifying cognitive and affective shifts in perception and to clarify ambiguities around the contradictory findings of previous pupillometry studies regarding fluency effects and response times in art research (Kuchinke et al., 2009; Elschner et al., 2018). These studies focused solely on maximum dilation and end-of-trial pupil responses, overlooking other potential indicators of cognitive and affective processes throughout the trial period. We expanded the analysis and considered (1) the timing of maximum and (2) the average dilation throughout the entire trial period. This comprehensive approach allowed us to investigate whether some noteworthy pupil responses might have been missed in previous studies and whether late physiological responses correlate with behavioral responses (Laeng et al., 2012; Mathôt, 2018).

For the recognition task (part 1), neither stimulus content nor accessibility significantly impacted average pupil size. In contrast, the time of maximum pupil dilation presented a different pattern: lower fluency predicted earlier maximum dilation, while face stimuli and correct response trials corresponded with later maximum dilation. During free viewing (part 2), average pupil dilation significantly responded to our main factors, whereas the time of maximum dilation did not. High-fluent artworks and face stimuli led to larger pupil dilation.

When comparing parts 1 and 2, part 2 showed significantly higher average pupil dilation, and the impact of accessibility and stimulus content was more pronounced in part 2. Although these results hint that average pupil dilations might be a marker for artwork evaluations, or emotional response, the found results on ratings are still inconclusive: average pupil dilation correlated especially with high clearness and negative valence. Liking showed a light positive, while complexity had a negative association with dilations. These results add to the controversial findings of prior studies (e.g., Kuchinke et al., 2009; Elschner et al., 2018), leaving, still, many open questions, if pupil dilations can truly represent directionality of emotional response, or, just an emotional response *per se*—might this be a positive, arousing, or negative one.

In addition, our findings contrast with previous studies that focused solely on pupil size at the end of trials (Kuchinke et al., 2009; Elschner et al., 2018). By examining the entire trial period, we identified that maximum pupil dilation occurred at both the beginning and end of trials in part 1 (see Figure 7). This pattern indicates two distinct phases of participants’ physiological responses. The initial dilation at the start of the trial likely signifies the engagement of a search pattern, as the brain allocates resources to scan and process new information. This phase is closely tied to the adaptive gain theory, which posits that the locus coeruleus–norepinephrine (LC-NE) system in the brain modulates cognitive functions to optimize performance (for further reading adaptive gain theory considering locus coeruleus activity, Gilzenrat et al., 2010; Jepma and Nieuwenhuis, 2011). According to this theory, the LC-NE system dynamically adjusts between the exploitation of recognizing familiar information and the exploration of new stimuli. The initial pupil dilation may thus reflect an increased LC activity, gearing the cognitive system toward exploration and heightened attention. Conversely, the dilation observed at the end of the trial could indicate recognition processes, where the LC-NE system shifts toward exploiting known information. This cycle of dilation aligns with the adaptive gain theory’s framework, suggesting that the LC-NE system’s activity is crucial in modulating attention and cognitive effort in response to changing task demands.

In sum, although we did find clear differences in pupillary measures considering recognition, i.e., time of maximum dilation, and as a potential affective response, the average pupil dilation, further research is necessary to deepen the understanding of our research. Future research could consider locus coeruleus activity (Aston-Jones and Cohen, 2005; Laeng et al., 2012; Larsen and Waters, 2018; Spee et al., 2018) during Gestalt recognition tasks and Aha moments to further explore the interplay of bottom-up and top-down influences as well as search patterns (seeking for meaning) relevant for art appreciation. Considering emotional response, we would suggest that pupillary measures should be connected with other physiological and/or neuroscientific approaches, as interpreting our results in relation to prior research results does not show a clear picture.

### 5.3 Limitations and considerations

Several limitations and considerations warrant further discussion in our study. Primarily, our attempt to manipulate effort motivation through a private versus public condition failed to produce the anticipated impact. It could be that our manipulation was inadequate, or alternatively, it suggests that the act of identifying Gestalt in art, a socially and culturally trained behavior (Fingerhut, 2020, 2021; Spee et al., 2022), does not require further extrinsic motivation. Furthermore, the public condition might have been less compelling as art interaction often hinges on individual interpretation and personal meaning, rendering the opinions of others less influential.

Our results point toward a delayed response in achieving the Aha moment. While it might be questioned whether part 1 truly reflected an art interaction or merely a recognition challenge, we contend that art inherently poses a challenge—discerning patterns in ambiguity, interpreting the artist’s intent, and crafting our personal interpretations. Thus, our design might not diverge significantly from a realistic encounter with art, considering that art engagement often entails giving a thorough interpretation, an act that may enhance one’s societal status (Spee et al., 2022) through perceived intelligence.

We acknowledge that our analysis connecting part 1 and part 2, specifically that performance outcome influenced the ratings made in part 2, could be questioned. However, maintaining research in cognitive theories (Schwartenbeck et al., 2013; Kesner, 2014; Seth, 2019) and our own experiences interacting with art suggest that success or failure in comprehending art, along with the context, influence our judgments. Given the short time span of part 1 (a few minutes), we support our exploratory findings, suggesting that the two parts influenced each other. Furthermore, the sequential nature of the experiment itself might have created a carry-over effect, where the cognitive processes and performance choices made in part 1 could have shaped the subjective ratings in part 2.

Certainly, we are aware that the rapid serial presentation of the artworks in a dark laboratory room influenced pupillary measurements. This is an inherent limitation of the study design. However, the approach was successfully employed in previous research (Laeng et al., 2011, 2012) and, indeed, our findings still yielded significant results. However, acknowledging this constraint is essential and points toward the potential for alternative methodologies that may yield more naturalistic and comprehensive data. The advent of new technologies, such as movable eye trackers or brain pattern measures that allow participants more freedom of movement, could provide a more immersive and realistic environment for observing visual art interaction. This, in turn, could help to enhance the ecological validity of future studies, making findings more applicable to real-world art contexts.

Finally, as a significant limitation, it is important to note that our study, like previous research, was unable to fully disentangle whether pupil dilations were indicative of recognition or merely affect, as both are potential triggers for such physiological responses. We did find that different pupillary measures appear relevant for the diverse attentional states. Nonetheless, future research may endeavor to find more precise ways to separate these two effects to gain a clearer understanding of the cognitive and emotional processes involved in art appreciation.

### 5.4 Summary and research prospects

Our study revealed that accurate or inaccurate predictions of Gestalt significantly influenced the time of behavioral response, that is of stating to have gained an Aha moment, suggesting that commonly observed behaviors, such as fluency effects, can be manipulated—or even emphasized—depending on the art task presented or participants state attitude. This suggests that the act of evaluating an artwork, the subsequent experience, the exploration process, and the desired outcomes are all products of a dynamic, reciprocal process (Van de Cruys and Wagemans, 2011b; Van Geert and Wagemans, 2020). Our results illuminate the complex ways in which we interact with visual art, showing how the delicate interplay of Gestalt recognition, insight, and exploration guides Aha and, potentially, our art judgments and emotional response to art.

Crucially, our findings demonstrate that Gestalt recognition as a nuanced process can be measured both behaviorally and physiologically, exemplified through our use of pupillometry as a measure of cognitive shifts. Given that pupillometry correlates with brain state (Aston-Jones and Cohen, 2005; Gilzenrat et al., 2010; Laeng et al., 2012; Larsen and Waters, 2018; Mathôt, 2018; Jefferies and Di Lollo, 2019), we propose future research in art appreciation and neuroaesthetics to consider task-evoked differences in locus coeruleus activity during interaction with visual art (Spee et al., 2018). We also underscore the importance of considering Gestalt recognition in the process of art evaluation, whether it concerns familiar Gestalt patterns from past experiences or novel figure-pattern constellations (Kesner, 2014; Van de Cruys et al., 2017; Fingerhut, 2020).

Building on seminal theories positing that piecing together disparate features into a unified Gestalt and assigning meaning to the recognized pattern is an inherent aspect of art viewing (Wertheimer, 1923; Arnheim, 1954), we argue that the ability to predict Gestalt is an integral part of the art experience. It not only guides cognitive and affective processing but also profoundly shapes the overall quality of art experiences. We propose that the rewards derived from viewing art are not solely contingent on personal taste or stimulus valence but are intimately tied to the capacity for accurate Gestalt prediction. In this light, both the quality of the Gestalt and the precision of the predictions play a crucial role in determining the pleasure derived from art appreciation. This pivotal finding suggests a new dimension for future investigations into the cognitive and emotional landscapes of art interactions. Accordingly, both ‘good Gestalt’ and ‘good predictions’ would determine how rewarding the act of viewing an artwork can be.

## Data availability statement

The original contributions presented in the study are included in the article/Supplementary material; further inquiries can be directed to the corresponding author.

## Ethics statement

The studies involving humans were approved by Ethics committee of the University of Vienna. The studies were conducted in accordance with the local legislation and institutional requirements. The participants provided their written informed consent to participate in this study.

## Author contributions

BS development of study design, main lead in the design of analysis, collected data, contributed data and analysis tools, performed analysis, and wrote the main manuscript. JA analysis, especially eye-tracking data. JM analysis, especially behavioral data. UT: conceived and designed the analysis and supervision of data analysis. MP contributed to the analysis. HL supervised development of study design. All authors contributed to the article and approved the submitted version.

## Footnotes

^1^The pupil is controlled by the iris sphincter muscle, constricting the pupil and the dilatory pupil muscle that promotes dilation (Borgdorff, 1975). Pupils respond to brightness or changes in depth of the focal field (mediated by cholinergic activity). Reduced illumination can provoke dilation up to 120% size compared to standard light conditions (Wyatt, 1995). Pupils can also constrict or dilate due to arousing stimuli or mental effort (Mathôt, 2018). Task-evoked psychologically triggered pupil reactions of affective and cognitive quality show modest effects; on average, the pupil diameter changes about 0.5 mm (≈ 20%, Beatty and Lucero-Wagoner, 2000; Laeng et al., 2012). Pupil responses, therefore, are also a measure of higher cognitive function, though they are mostly reflexive and involuntary (Mathôt, 2018).
