# Supplementary material for: The dynamics of experiencing Gestalt and Aha in cubist art: pupil responses and art evaluations show a complex interplay of task, stimuli content, and time course
Source: Front Psychol. 2024 Mar 13;15:1192565. doi: 10.3389/fpsyg.2024.1192565 (PMC10966367; doi:10.3389/fpsyg.2024.1192565)
Supplement: Supplementary file 1 [file Data_Sheet_1.pdf]

Supplementary Material

The Dynamics of Experiencing Gestalt and Aha in Cubist Art: Pupil Responses and Art Evaluations Show a Complex Interplay of Task, Stimuli Content, and Time Course

Blanca T.M. Spee[1,2,3*], Jozsef Arato[1], Jan Mikuni[2], Ulrich S. Tran[2], Matthew Pelowski[1,2], Helmut Leder[1,2]

[1]Vienna Cognitive Science Hub, University of Vienna, Vienna, Austria

[2] Department of Cognition, Emotion, and Methods in Psychology, Faculty of Psychology, University of Vienna, Vienna, Austria

[3] Radboud University Medical Center, Donders Institute for Brain, Cognition and Behavior, Department of Neurology, Center of Expertise for Parkinson & Movement Disorders, Nijmegen, The Netherlands

1 Supplementary Tables

Table S1
Full list of stimulus-set rated including name of artist, title, year, stimulus content, and degree of fluency. Note, English translation if existing. NA = year not available.

Artist	Title	Year	Stimulus content	Degree of fluency
Albert Gleizes	Man on a Balcony	1912	Faces	high
Juan Gris	Portrait of Pablo Picasso	1912	Faces	high
Pablo Picasso	Woman with Mustard Pot	1910	Faces	high
Pablo Picasso	Woman with a Fan	1907	Faces	high
Pablo Picasso	Femme au bouquet	1909	Faces	high
Pablo Picasso	La Reine Isabeau	1909	Faces	high
Pablo Picasso	Portrait of Ambroise Vollard	1910	Faces	high
Fernand Leger	The Sitted Woman	1914	Faces	medium
Juan Gris	Portrait of Madame Josette Gris	1916	Faces	medium
Pablo Picasso	La danse aux voiles	1907	Faces	medium
Pablo Picasso	The Dryad	1908	Faces	medium
Unknown	Unknown	NA	Faces	medium
Georges Braque	Woman with a Guitar	1913	Faces	low
Ljubow Sergejewna Popowa	Composition with Figures	1915	Faces	low
Pablo Picasso	Three Women	1908	Faces	low
Pablo Picasso	Daniel-Henry Kahnweiler	1910	Faces	low
Unknown	Unknown	NA	Faces	low
Unknown	Unknown	NA	Faces	low
Unknown	Unknown	NA	Faces	low
Unknown	Unknown	NA	Faces	low
Albert Gleizes	La Cathédrale de Chartes	1912	Landscapes	high
Georges Braque	Landscape with houses	1909	Landscapes	high
Georges Braque	Le Viaduc de l'Estaque	1908	Landscapes	high
Georges Braque	Houses at L'Estaque	1908	Landscapes	high
Pablo Picasso	Paysage aux deux figures	1908	Landscapes	high
Pablo Picasso	Landscape with bridge	1909	Landscapes	high
Georges Braque	The Castle of La Roche-Guyon—2	1909	Landscapes	medium
Georges Braque	The Castle of La Roche-Guyon—3	1909	Landscapes	medium
Georges Braque	L'Estaque	1908	Landscapes	medium
Jean Metzinger-Paysage	Le Village	1911	Landscapes	medium
Josef Čapek	Továrna	1912	Landscapes	medium
Pablo Picasso	La Rue des Bois	1908	Landscapes	medium
Pablo Picasso	Le Sacré-Coeur	1910	Landscapes	medium
Pablo Picasso	The Reservoir	1909	Landscapes	medium
Georges Braque	La Sacre Coeur	1910	Landscapes	low
Georges Braque	The Castle of La Roche-Guyon—1	1909	Landscapes	low
Georges Braque	Fishing Boats	1909	Landscapes	low
Pablo Picasso	Le port de Cadaqués	1910	Landscapes	low
Unknown	Unknown	NA	Landscapes	low

Table S2
Descriptive analysis of response times in ms and separated for each condition.

	public		private	
	**Mean**	**SD**	**Mean**	**SD**
faces	505.08	85.32	506.46	92.19
landscapes	434.61	91.51	440.30	92.78
high-fluent	510.09	87.86	513.45	98.40
medium-fluent	451.14	107.25	449.62	90.79
low-fluent	449.96	100.06	457.41	96.00
total response time	469.79	78.84	472.89	73.07

Table S3
Part 1, LMMs for average pupil size all predictors. Dependent variable was average pupil dilation. Independent variables were accessibility, stimulus content, trail number, performance outcome, and effort motivation.

			95% CI			
Fixed effects	Estimate	SE	Lower	Upper	*z*-value	Pr(>|z|)
Intercept	-65.91	15.21	- 95.72	-36.11	-4.33	<.00
accessibility	1.53	3.93	-6.18	9.23	0.39	.70
stimulus content	4.98	6.11	-7.00	16.95	0.81	0.42
trial	-0.71	0.34	-1.38	-0.04	-2.06	.04
performance outcome	-3.41	8.17	-19.43	12.61	-0.42	0.68
effort motivation	-20.77	11.63	-43.57	2.020	-1.79	0.07

Table S4
Part 1, LMMs for maximum pupil size all predictors. Dependent variable was time of the maximum pupil dilation. Independent variables were accessibility, stimulus content, trail number, performance outcome, and effort motivation.

			95% CI			
Fixed effects	Estimate	SE	Lower	Upper	*z*-value	Pr(>|z|)
Intercept	0.26	0.04	0.18	- 0.34	6.06	<.001
accessibility	-0.04	0.01	-0.06	-0.02	-4.03	<.001
stimulus content	0.10	0.02	0.07	0.13	6.54	<.001
trial	0.00	0.00	0.00	0.00	4.17	<.001
performance outcome	0.07	0.02	0.03	0.11	3.44	.001
effort motivation	-0.02	0.04	-0.09	0.06	-0.42	0.67

Table S5
Effects of Part 1 on Part 2; fixed effects in the LMM predicting ratings; dependent variable is represented by the ratings; independent variable was performance-outcome with 'correct' as baseline; we included the intercept as a random coefficient (the intercept could vary between participants).

		95% CI			
Fixed effects	Estimate	Lower	Upper	*t*-value	Pr(>|*t*|)
**Complexity**					
Intercept	4.34	4.21	4.47	64.45	*p*<.001
False-alarm	0.18	-0.08	0.45	1.37	0.17
Miss	0.51	0.34	0.68	6.01	*p*<.001

**Comprehension**					
Intercept	3.90	3-75	4.05	51.08	*p*<.001
False-alarm	-0.17	-0.43	0.11	-1.18	0.237
Miss	-1.18	-1.36	-1.00	-13.14	*p*<.001
**Valence**					
Intercept	4.08	3.98	4.17	79.92	*p*<.001
False-alarm	0.40	0.15	0.65	3.16	*p*<.01
Miss	-0.27	-0.43	-0.12	3.39	*p*<.001
**Arousal**					
Intercept	3.93	3.81	4.04	65.77	*p*<.001
False-alarm	-0.19	-0.46	0.07	1.43	0.15
Miss	0.44	0.22	0.61	5.09	*p*<.001
**Clearness**					
Intercept	3.35	3.23	3.47	54.50	*p*<.001
False-alarm	-1.55	-2.00	-1.10	6.89	*p*<.001
Miss	-0.43	-0.72	-0.15	2.97	*p*<.01
**Liking**					
Intercept	4.11	3.97	4.24	61.25	*p*<.001
False-alarm	0.42	0.14	0.71	2.89	*p*<.01
Miss	-0.54	-0.73	-0.36	5.81	*p*<.001

2 Supplementary Figures Legend

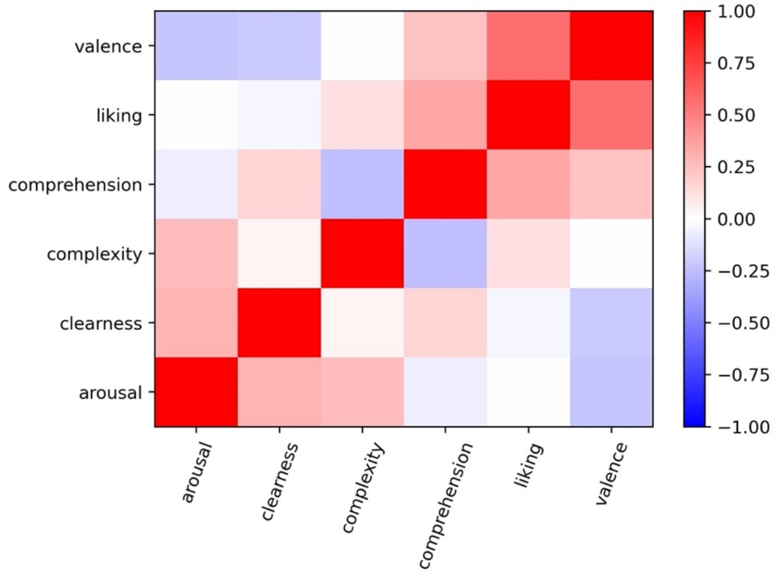

Figure S1. Heatmap pearson correlation between each of the ratings, part 2.

a. b.

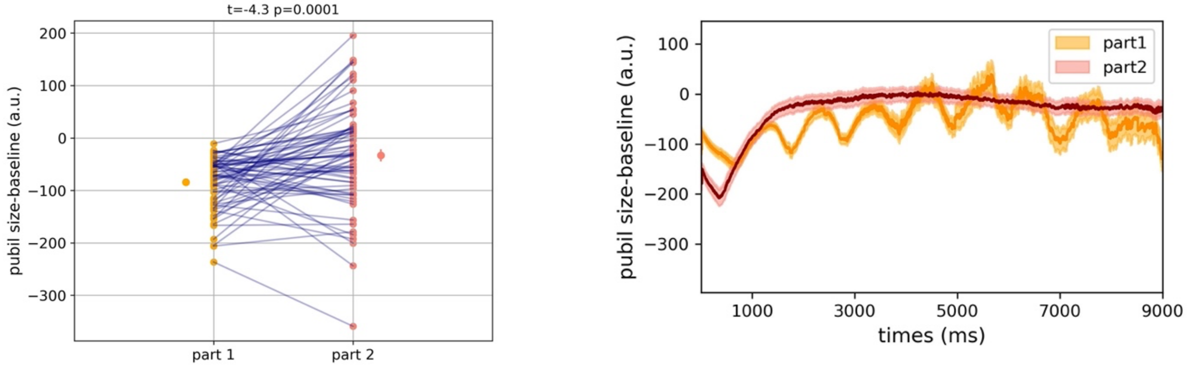

Figure S2. Baseline-corrected average pupil dilation, part 1 and part 2.

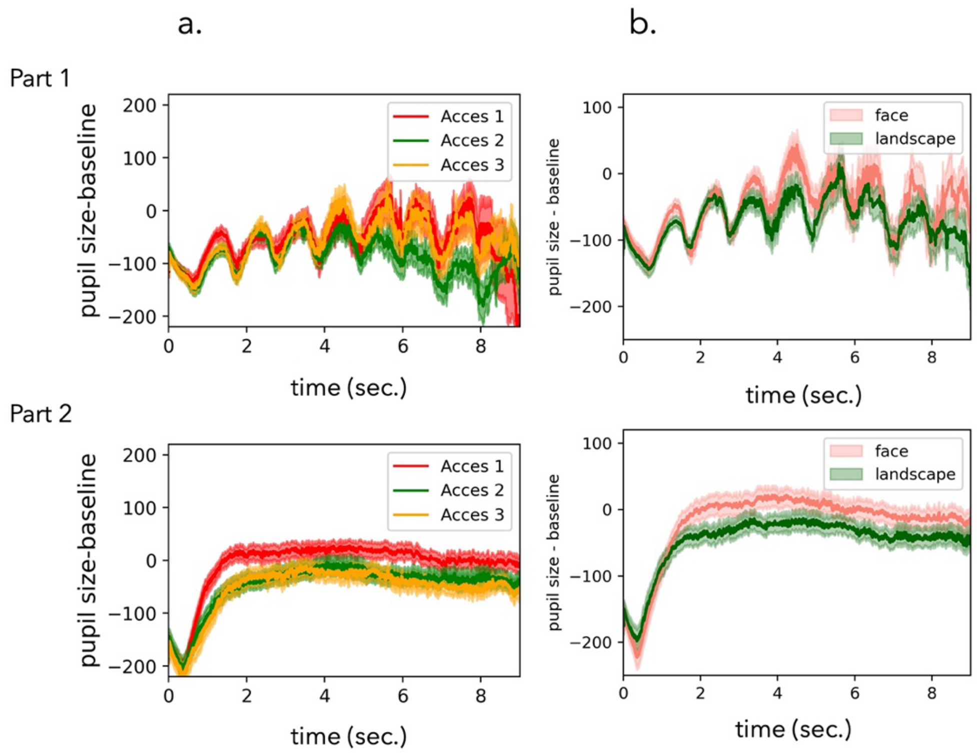

Figure S3. Baseline-corrected average pupil dilation, raw data, part 1 and part 2.
